# Hybrid thermosensitive-mucoadhesive *in situ* forming gels for enhanced corneal wound healing effect of L-carnosine

**DOI:** 10.1080/10717544.2021.2023236

**Published:** 2022-01-24

**Authors:** Zeinab Fathalla, Wesam W. Mustafa, Hamdy Abdelkader, Hossam Moharram, Ahmed Mohamed Sabry, Raid G. Alany

**Affiliations:** a Pharmaceutics Department, Faculty of Pharmacy, Minia University, Minia, Egypt; b Department of Chemical and Pharmaceutical Sciences, Kingston University London, Kingston upon Thames, UK; c Department of Pharmacy, Al-Mustafa University College, Baghdad, Iraq; d Pharmaceutics Department, Faculty of Pharmacy, Deraya University, New Minia, Egypt; e Ophthalmology Department, Faculty of Medicine, Minia University, Minia, Egypt; f Drug Discovery, Delivery and Patient Care Theme, Faculty of Science, Engineering and Computing, Kingston University, Kingston upon Thames, UK; g School of Pharmacy, The University of Auckland, Auckland, New Zealand

**Keywords:** L-carnosine, *in situ* gel-forming, cornea, ocular delivery, gelation time and temperature, texture analysis

## Abstract

**Purpose:**

Thermosensitive *in situ* gels have been around for decades but only a few have been translated into ophthalmic pharmaceuticals. The aim of this study was to combine the thermo-gelling polymer poloxamer 407 and mucoadhesive polymers chitosan (CS) and methyl cellulose (MC) for developing effective and long-acting ophthalmic delivery systems for L-carnosine (a natural dipeptide drug) for corneal wound healing.

**Methods:**

The effect of different polymer combinations on parameters like gelation time and temperature, rheological properties, texture, spreading coefficients, mucoadhesion, conjunctival irritation potential, *in vitro* release, and *ex vivo* permeation were studied. Healing of corneal epithelium ulcers was investigated in a rabbit’s eye model.

**Results:**

Both gelation time and temperature were significantly dependent on the concentrations of poloxamer 407 and additive polymers (chitosan and methyl cellulose), where it ranged from <10 s to several minutes. Mechanical properties investigated through texture analysis (hardness, adhesiveness, and cohesiveness) were dependent on composition. Promising spreading-ability, mucoadhesion, transcorneal permeation of L-carnosine, high ocular tolerability, and enhanced corneal epithelium wound healing were recorded for poloxamer 407/chitosan systems.

**Conclusion:**

*In situ* gelling systems comprising combinations of poloxamer-chitosan exhibited superior gelation time and temperature, mucoadhesion, and rheological characteristics suitable for effective long-acting drug delivery systems for corneal wounds.

## Introduction

The cornea is a transparent ocular tissue at the front of the eye. The cornea has both protective and refractive functions. The normal structure and function of the cornea can be adversely affected by many factors, such as trauma, surgery, and applied ocular drugs (Ljubimov & Saghizadeh, 2015). The ultimate outcome is corneal ulcers and corneal blindness if left untreated. The available treatment options are limited to ocular lubricant and antibiotics without an effective drug therapy that treats corneal wound healing. L-carnosine is a native dipeptide biosynthesized from β-alanine and l-histidine through carnosine synthase (Mendelson, 2000). L-carnosine is an antioxidant commonly found in human tissues, such as muscles and brain (Cao et al., 2021).

Recent reports highlighted its benefits for treating age-related ocular diseases, such as cataracts and corneal disorders. This is because of three main favorable characteristics that have been attributed to this dipeptide drug (Litwack, 2018). L-carnosine has antioxidant effects, metal chelating, and antiglycation properties (Turner et al., 2021). These features protect aging ocular tissues from oxidative stress, glycation, and post-translational modification of structural (lens crystallins) as well as functional (enzymes) proteins in the human eye (Babizhayev et al., 2009, 2002). Recent reports highlight the role of L-carnosine as a potential anticancer agent (Gaafar et al., 2021; Turner et al., 2021). Pegylated liquid crystalline nanoparticles loaded with L-carnosine have been investigated for superior antitumor activities compared to L-carnosine alone and L-carnosine phytosomes (Gaafar et al., 2021).

L-carnosine has been reported to promote corneal wound healing without scar formation. These wound healing properties are attributed to repairing impaired metabolism in the cornea by protecting native proteins in the corneal tissues from oxidative damage and modulating the inflammatory responses (Quinn et al., 1992).

There are scarce reports on the development of eye formulations for L-carnosine; however, the more lipophilic prodrug derivative of N-acetyl carnosine has been patented in the USA and is available as eye drops containing 1% N-acetyl carnosine (Can-C^®^). N-acetyl carnosine undergoes biotransformation into L-carnosine upon topical ocular administration (Babizhayev et al., 2009).

Preformulation studies on the active form L-carnosine indicated that the drug has considerable chemical stability and a log *p*-value of ∼.03. Therefore, L-carnosine has balanced hydrophilicity-hydrophobicity attributes where permeation through the lipophilic corneal barrier is likely to be the rate-determining step in its ocular absorption (Abdelkader et al., 2015).

*In situ* gelling drug delivery systems (also called gel-forming systems) can offer several advantages over preformed gels. *In situ* gels are suitable for simple and scalable preparation; they offer the convenience of being administered as free-flowing solution eye drops; and converted to gel on the ocular surface. The *in-situ* gels retain the drug at the elected superficial region and potentially reduce the frequency of administration (Cassano et al., 2021). In this respect, poloxamers have tissue penetration enhancing properties that could facilitate hydrophilic drugs like L-carnosine for accessing the corneal lipid barrier. Poloxamer 407-based hydrogels have been investigated for mucoadhesive properties, time-release properties, and tissue tolerability (Zhang et al., 2015; Giuliano et al., 2018). Chloramphenicol (antibiotic) *in situ* gels for ocular delivery were based on the combination of poloxamer 407 and hydroxypropyl methyl cellulose; they showed optimized viscosity, pH, and gelling capacity (Kurniawansyah et al., 2020). Poloxamer belongs to a unique synthetic non-ionic polymer with surface-active properties due to containing an inner hydrophobic core of poly (propylene oxide) and outer hydrophilic chains of poly (ethylene oxide). Poloxamers have favorable physiological properties, such as thermal-dependent gelation and self-assembly as well as acceptable biocompatibility, high drug-loading capacity, and tissue tolerability that renders poloxamer-based gels promising drug delivery systems (Zarrintaj et al., 2020, Carvalho et al., 2021). Poloxamers can be considered safe for both oral and dermal application with LD 50% ∼5 g/kg and when applied one daily for 14 days, no skin erythema or sensitization times were recorded (Carvalho et al., 2021).

However, thermal gelation usually occurs at a relatively high concentration of poloxamers (≥15% w/w) at physiological eye surface conditions. This might pose toxicological and irritation concerns to the ocular tissues. In addition, the onset of gelation of poloxamer alone varies from seconds to minutes which would be long enough for significant drug loss (Fathalla et al., 2017). These conditions might not suit the dynamic properties on the surface of the eye from frequent blinking and rapid turnover of tear fluid (Lang et al., 2002). This retardation of sol-to-gel transition may likely lead to drug loss of the instilled dose due to the rapid dilution by precorneal tear and reflex tearing. To the best of our knowledge, there are limited reports on hybrid gels comprising poloxamers combined with mucoadhesive polymers for optimized ocular drug delivery in terms of rheological, mechanical, mucoadhesion, spreading ability, and overall ocular safety and efficacy.

The goal of this work was to explore the possibility of combining poloxamer with other mucoadhesive polymers, such as chitosan (CS) and methylcellulose (MC) in an attempt to develop an *in-situ* gelling formulation with optimized gelation time, temperature muco-adhesive characteristics, and improved corneal wound healing properties. Gelation of poloxamer mainly relies on micelles packing and entanglement (Cabana et al., 1997). The inclusion of drugs or additives has been reported to interfere with micelles formation, and subsequently, interfere with the sol-to-gel transition temperature (Tsol-gel) (Edsman et al., 1998).

This study reports on the effects of CS and MC on Tsol-gel of the poloxamer 407 used. The *in-situ* gelling poloxamer-based preparations loaded with L-carnosine (LC) were evaluated for their mechanical rheological spreading as well as mucoadhesive properties. *Ex-vivo* permeation studies were carried out to establish the possibility of using *in situ* gelling combinations of poloxamer-MC and poloxamer-CS as novel hybrid polymeric carrier systems for LC to the ocular surface.

## Materials

L-carnosine (LC), poloxamer 407 (P407, culture tested), chitosan (CS) high molecular weight (Brookfield viscosity 800,000 CP), methyl cellulose (MC), porcine mucin, and benzalkonium chloride (BKC) were purchased from Sigma Aldrich, the UK.

## Preparations of *in situ* gels

P407 solution was prepared using the cold method. In brief, accurately weighed amounts of the polymers were added to cold aqueous solutions of CS or MC that were set at 4 °C (as shown in Table 1). The polymer solutions were kept in a cold room for 24 h under constant stirring to ensure complete dissolution. CS was dissolved in acetic acid (1% v/v) and the final pH of CS solution was raised to 5.5 using an aqueous solution of sodium hydroxide (1 M). For drug-loaded gels, LC was added and dissolved in CS or MC solutions, then the P407 was finally dissolved to form final LC (1% w/v)-loaded *in situ* gels.

**Table 1.** Composition of the prepared *in situ* gels, gelation time, gelation temperature and pH.

No.	Formulation*	P407 (%w/v)	CS/MC (%w/v)	Gelation temperature (°C)	Gelation time (s)	pH
1	F-P1	14	–	40.0 ± 0.9	Over 7 min	6.21 ± 0.01
2	F-P2	16	–	32.5 ± 0.5	30.2 ± 5.6	6.90 ± 0.02
3	F-P3	18	–	34.1 ± 0.9	20.3 ± 1.9	7.2 ± 0.14
4	F-P4	20	–	30.3 ± 0.5	12.3 ± 5.1	7.1 ± 0.22
5	F-P5	22	–	29.0 ± 1.0	11.4 ± 3.0	6.80 ± 0.33
6	F-P6	25	–	25.0 ± 0.5	9.2 ± 1.7	7.25 ± 0.05
7	F-P7	30	–	20.5 ± 0.5	7.5 ± 2.3	7.30 ± 0.22
8	F-P2/CS0.5	16	0.5	31.1 ± 1.5	55.2 ± 8.5	6.04 ± 0.13
9	F-P2/CS1	16	1	30.2 ± 2.8	33.1 ± 2.0	6.07 ± 0.07
10	F-P2/CS1.5	16	1.5	29.1 ± 1.3	30.8 ± 2.5	6.00 ± 0.02
11	F-P3/CS0.5	18	0.5	32.0 ± 0.5	17.9 ± 3.5	6.15 ± 0.23
12	F-P3/CS1	18	1	34.5 ± 0.5	13.5 ± 2.4	6.10 ± 0.01
13	F-P3/CS1.5	18	1.5	31.0 ± 1.5	12.5 ± 2.8	6.01 ± 0.11
14	F-MC0.5	–	0.5	–	Over 6 min	6.03 ± 0.02
15	F-MC1	–	1	–	Over 6 min	6.55 ± 0.04
16	F-MC1.5	–	1.5	–	Over 6 min	6.77 ± 0.21
17	F-P2/MC0.5	16	0.5	34.0 ± 0.5	22.6 ± 3.3	7.07 ± 0.01
18	F-P2/MC1	16	1	31.0 ± 0.5	20.1 ± 1.2	7.00 ± 0.02
19	F-P2/MC1.5	16	1.5	24.0 ± 1.00	44.5 ± 2.7	6.90 ± 0.22
20	F-P3/MC0.5	18	0.5	34.0 ± 1.3	13.2 ± 1.7	7.10 ± 0.11
21	F-P3/MC1	18	1	33.0 ± 0.99	15.3 ± 2.1	7.22 ± 0.04
22	F-P3/MC1.5	18	1.5	24.0 ± 1.5	45.8 ± 5.1	7.40 ± 0.05

Data are expressed as mean values ± *SD* (*n* = 3).

*All formulations contained L-carnosine with a concentration of 1% w/v.

## Physicochemical characterization of formulations

### Gelation time and gelation temperature

The time required for the onset of gelation and subsequent transition from sol-to-gel was called gelation time. This parameter was recorded employing aluminum pans that were mounted on a hot plate prewarmed to 35 °C. Once the aluminum pan was hot enough, a few drops of the test formulations (Table 1) were placed on the hot pan using a micropipette. The aluminum pan was tilted at a right angle (90°) to see if the formulation has turned into the gel or still liquid. The final gelation time is the point at which the instilled formulation drops became thick and ceased moving upon tilting. A stopwatch was used to record the time of gelation. The same procedure was repeated for all the prepared *in situ* gelling formulations and the results were presented as the average of triplicate samples (*n* = 3).

The temperature at which the sol-gel transition occurred was called gelation temperature (Tsol-gel). This temperature was recorded using the visual tube inversion method. Each formulation was kept at fridge temperature (4–8 °C), transferred into a glass test tube; a thermometer was placed in the test solutions left at ambient conditions; once raised to the room temperature, the test tube was transferred into a water bath at a temperature of 25 ± 1 °C. The temperature was gradually raised at a rate of 1 °C/min and the temperature at which gelation occurred (the surfaces remained immobile by tiling the tubes to the horizontal position) was recorded (Ur-Rehman et al., 2011).

### Rheological characteristics

The viscosity of the developed *in situ* gels was determined at different rotational speeds (10–100 rpm) and constant temperature using a rotational viscometer (Brookfield DV-II, Essex, UK) equipped with spindle 62.

### Texture analysis

**Figure 1.**
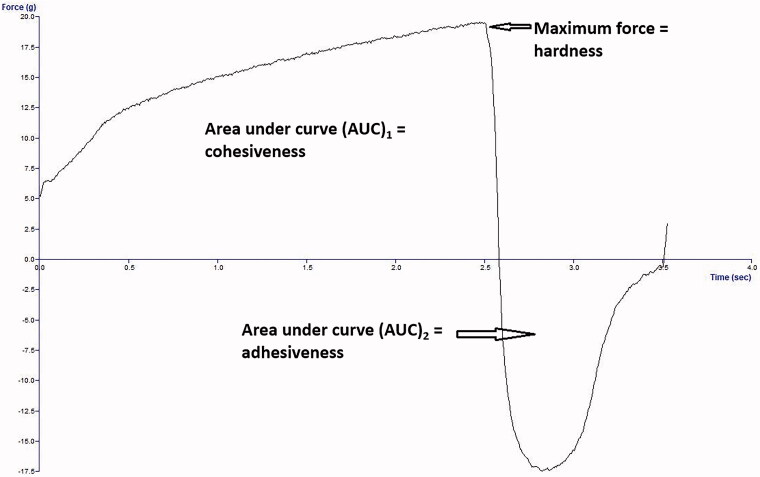
Representative texture analysis profile (force *vs.* time plot) to demonstrate the three parameters measured (hardness, cohesiveness, and adhesiveness).

Mechanical properties of the prepared *in situ* gels were studied using a TA-XT-plus Texture Analyzer (Stable micro-Systems, Surrey, the UK) as previously reported (Fujimoto et al., 2016). Sample formulations (35 g each) were placed in 50-ml glass beakers. An analytical probe (1 cm diameter) was immersed twice in each gel sample at a predetermined rate and depth of 1 mm/s and 10 mm, respectively, allowing a delay period of 10 s between each immersion. The maximum force required to penetrate to that depth is called gel strength. Measurements were performed at two temperatures (4 and 35 °C). From the force–distance curve created by the Texture Exponent 32 software; the following texture parameters were estimated:

Gel strength (hardness) is the maximum force (mN) of the positive peak; cohesiveness is the area under the curve (AUC)_1_ of the positive area in mN·mm; adhesiveness is (AUC)_2_ of the negative area in mN·mm, as shown in Figure 1.

### Spreading-ability of L-carnosine loaded formulations

#### Contact angle and spreading coefficient

Contact angle (*θ*) is the angle formed where the liquid-vapor interface meets the solid surface. This was experimentally determined by using a drop shape analyzer (Kruss Drop Shape Analysis, Hamburg, Germany).

Complete wetting of the solid surface is achieved when *θ* is equal to zero. Wetting in which a liquid spreads over the solid surface is known as spreading. The tendency of spreading can be assessed by determining the spreading coefficient (*S*) as expressed by Equation (1) (Florence & Attwood, 1998):

(1) S = γ (cos θ – 1)

Where *S* is the spreading coefficient, *γ* is the tension the surface tension of the liquid placed onto the solid substrate and *θ* is the contact angle.

The γ values for L-carnosine *in situ* gels were determined using a Torsion balance (Malvern Wells, UK).

### Ocular irritation studies

#### HET-CAM

The *in vitro* ocular irritation based on modified hen’s egg chorioallantoic membrane (HET-CAM) assay was adopted to investigate the conjunctival irritation of selected *in situ* gels (Abdelkader et al., 2012). Fertilized White Leghorn eggs were incubated at temperature and relative humidity of 37.5 ± 0.5 °C and 66 ± 5%, respectively for 3 days. After 3 days of incubation, the eggshells were opened by cracking and the content was poured into growing Petri dishes. The yolk sacs were examined for any visible rupture. Living embryos with an intact yolk sac were incubated further and utilized for the irritation investigation assay. The following samples were used:

Sodium hydroxide (1 M) as positive control; propylene glycol as mild-to-moderate irritant control; saline was used as a negative control. These three controls were employed for validation purposes.

Once a test formulation is placed on the CAM a time-dependent numerical score was adopted for the signs of conjunctival irritation of hyperemia, hemorrhage, and clotting as described before (Abdelkader et al., 2012).

#### Mucoadhesion studies

**Figure 2.**
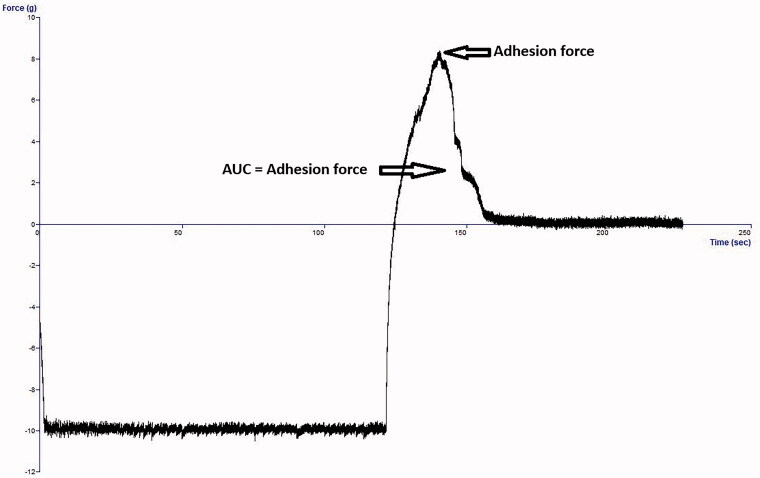
Representative mucoadhesion profile (force *vs.* time) using mucin disk detachment from the formulation.

Mucoadhesion of selected *in situ* gels was studied using the Texture analyzer (Stable micro-Systems, Surrey, the UK) as previously mentioned (Abdelkader et al., 2020). The specified amounts (0.25 g) of porcine mucin were compressed into 10 mm disks using an IR hydraulic press under a force of 10 tons for 30 s. The disks were fixed to the lower end of the Texture analyzer probe (10 mm in diameter) using a double adhesive tape. A sample of selected *in situ* gelling formulations equal to 25 g was pre-equilibrated at 35 °C in a water bath. The probe with mucin disk was gradually forced onto the gel surface. A force (5 g) was exerted for 3 min to ensure intimate contact between the mucin disk and the surface of the gel. The probe was pulled at a speed of 0.5 mm/s to a distance of 0.5 cm. The force (mN) needed to separate the disk from the gel was recorded and called the force of adhesion. Another mucoadhesion parameter called the work of adhesion (mN.mm) was estimated from the area under force (Figure 2) (Xu et al., 2014).

### Scanning electron microscopy

The surface of selected L-carnosine *in situ* gels (F-P3/CS0.5 and F-P3/CS1) was imaged and studied using SEM Carl Zeiss EVO 50, Cambridge, the UK. The microscope used was and equipped with a tungsten source and operated at an acceleration voltage equal to 10 KV. The surface of the gel was sputtered with gold.

### *In vitro* release

One ml aliquot of selected *in situ* gel formulations was transferred into the donor compartment of the Franz diffusion cells (Logan Instrument Corp., NJ, USA). The receptor compartment was filled PBS (12 ml) under stirring. Dialysis membrane (12–14 kDa molecular weight cut-off) separated the two compartments. The temperature was adjusted at 35 ± 0.5 °C. The amount of LC released was quantified using the HPLC method that was previously published elsewhere (Abdelkader et al., 2015). The HPLC system consisted of an isocratic mobile phase system (98% v/v: 2% v/v of trifluoro-acetic acid (0.1% v/v): acetonitrile with flow rate of 1 ml/min. a Supelcosil C18 column (5 µm; 25 × 0.46 cm, Supelco Corporation, PA, USA) at 40 °C; and UV detector set at 220 nm; and injection volume of 30 µl.

The cumulative release data were fitted into kinetics models (zero-order, first-order, and Higuchi Diffusional models) to elucidate drug release mechanisms from the selected gel formulations.

### Transcorneal penetration studies

The transcorneal permeation studies were performed using the Franz diffusion cells (Logan Instrument Corp., NJ, USA). The bovine eyes were collected from a local abattoir and were treated and dissected as previously described (Gaballa et al., 2020). The recipient compartment was filled with PBS (12 ml), and the donor compartment was filled with 1 ml of LC formulations. Two LC-loaded *in situ* gels (PCS12, PMC9) were studied. Drug solution (10 mg/ml) was used as a control. One ml of each sample equivalent to 10 mg/ml of LC was transferred into the donor compartment. The diffusion-cell system was maintained at 35 ± 0.5 °C. The amount of LC permeated across the mounted cornea (surface area 1.77 cm^2^) was analyzed by the HPLC method as described in the previous section.

The cumulative amounts of LC permeated were plotted against time and corrected for surface area. The apparent permeability coefficient (*P*_app_) was estimated through Equation (2):

(2) $$P_{\mathrm{app}}\;=\frac{F}{A\;Co}$$

Where *F* is the flux which is the slope of the cumulative drug permeation *vs.* time, *A* is the surface area and *Co* is the initial drug concentration.

### *In vivo* pharmacodynamic study (corneal ulcer induction and healing)

This study was approved by the Commission on the Ethics of Scientific Research under project code no. ES 10/2020, Faculty of Pharmacy, Minia University. of Ten rabbits, weighing between 1.5 and 2.0 kg, were grouped into two groups: group 1 received LC solution in their left eyes, and group 2 received F-P3/CS1 in their left eyes. The right eyes of both groups were left untreated and served as control.

Corneal ulcers were induced in both eyes using 70% ethyl alcohol by the alcohol delamination method as described before (Abdelkader et al., 2018). After induction of corneal ulcers, a single drop of each test formulation was instilled every 12 h for 3 days. Percentage (%) changes in ulcer size were determined using Equation (3):

(3) $$\text{\% Δ Ulcer size }=(\frac{D1-Dn}{D1})*100$$

where: *D*1 is the diameter of the ulcer at day 1, *Dn* is the diameter of the ulcer at day 2 or day 3.

### Statistical analysis

Flux, apparent permeability coefficients, and cumulative irritation scores were represented as mean values ± standard deviation (SD). Statistical analysis was performed using a one-way ANOVA; *p* < .05 and <.001 were considered statistically significant. Tukey’s pair-wise comparison was conducted and set at a 95% confidence interval. Analyses were performed using Graph Pad Software Version 3.05; San Diego, CA, USA.

## Results and discusssion

Twenty-two *t* hybrid *in situ* gelling formulations were prepared and studied with different poloxamer 407 (P) concentrations (14–30% w/v) and in combination with chitosan (CS) or methylcellulose (MC) using three different concentrations (0.5–1.5% w/v) of these two polymers. The prepared *in situ* gelling systems showed a pH range of 6–7.4 (Table 1). This indicates that the prepared gels were physiologically compatible with the ocular surface that which has pH in the range of 7.11 ± 1.5 (Lim et al., 2014).

### Gelation time and gelation temperature

Both gelation time and temperature (Tsol-gel) were recorded for the prepared *in situ* gel-forming systems (Table 1). It is obvious that both parameters were markedly dependent on the poloxamer 407 (P) concentration and the overall composition, where the changes in MC concentrations (0.5–1.5%) did not bring any observable changes in either gelation time and Tsol-gel. For example, the (Tsol-gel) were significantly (*p* < .05) lowered from 40 to 32 °C and the gelation time dramatically reduced from over 7 min to 30 s with changing the P concentration from 14% (F-P1) to 16% (F-P2), respectively.

Furthermore, hybrid formulations of poloxamer 407 at the optimized concentrations of 16% and 18% with three different concentrations (0.5, 1.0, and 1.5%) of CS did not markedly change the Tsol-gel; however, gelation time was reduced. F-P3-CS1 displayed a short gelation time (13.5 s) and Tsol-gel (34.5 °C) which is comparable to the physiological ocular surface temperature. Similar results were obtained for F-P3-MC0.5 with gelation time and temperature of 13 s and 34 °C, respectively.

The mechanism of thermal gelation of poloxamer is well-established. The temperature is the trigger that induces swelling of micelles comprising hydrated polyethylene oxide (PEO) chains at the outer hydrophilic shell and polypropylene oxide (PPO) chains in the inner hydrophobic core. Generally, increasing concentrations of poloxamers led to increasing the number of micelles and consequently reduces the gelation time and temperature. Similar results were reported elsewhere (Collaud et al., 2008).

Systems formulated with concentrations of poloxamer 407 below 14% w/v did not display gel characteristics at a temperature well above the body temperature and gelation time >7 min; on the contrary, concentrations >20% resulted in gel characteristics at ambient conditions. An increase of P concentrations >20% w/v reduced Tsol-gel to sub-physiological temperature and near ambient conditions (20–25 °C). Also, using relatively higher concentrations (1.5%) of MC obviously dropped the gelation temperature and prolonged gelation time. This was recorded on F-P2/MC1.5 and F-P3/MC1.5 compared to F-P2 and F-P3, where the gelation temperature was reduced by 10 °C (from 34 to 24 °C) and the gelation time was almost doubled from around 20 to >40 s.

Whilst shortening the gelation time is a desirable characteristic for an ophthalmic formulation; lowering of gelation temperature is not. This might promote undesirable gelation of the formulation at ambient temperature (20–25 °C) before instillation onto the surface of the eye with a temperature of around 35 °C.

Nevertheless, a shorter gelation time would be advantageous to reduce the time required for the instilled dose to transform into a viscous gel. Therefore, this could reduce the likelihood of the instilled dose to be rapidly diluted and lost *via* nasolacrimal drainage.

Formulations containing 0.5% w/v CS (F-P2/CS0.5) showed immediate gelation but reversed back to the ‘sol’ state after a few minutes. Increasing the concentration of CS may promote poloxamer entanglements and, thus, transition time becomes shorter and the erosion time of formed gel could be prolonged.

The gelation time recorded for *in situ* gelling formulations containing CS indicated that increasing CS concentration in the presence of P up to a certain limit, caused a significant decrease (*p* < .05) in the gelation time. For formulations F-P3/CS0.5 and F-P3/CS1, the gelation time was 17.9 ± 3.5 and 13.5 ± 2.4 s, respectively compared with formulation P3 formulation where gelation time was 20.3 ± 1.9 s (Table 1). However, increasing CS concentration up to 1.5% w/v did not have a significant (*p* > .05) influence on gelation time. This may be ascribed to the presence of CS in higher concentrations which increased the viscosity of the formulation and decreased gelation time. On the other hand, MC is a viscosity-enhancing agent which facilitates polymer chains entanglements into the P407-based formulations with a direct consequence in promoting more rapid conversion from sol to gel at a lower temperature as MC content increases.

Similar results were recorded for levofloxacin poloxamer 407 gels and levofloxacin gellan gum-poloxamer 407 hybrid gels. Both gelation time and temperature were dependent on poloxamer concentrations. The gelation temperature was 40 and 35 °C for 12 and 16% poloxamer 407 gels, respectively. Further, hybrid gels of gellan gum-poloxamer 407 reduced gelation temperature (36 °C) and prolonged gelation time (12 min), compared to gelation temperature (38) and gelation time (5 min) for only poloxamer 407 (14%)-based gel (Sapra et al., 2013).

### Rheological properties

**Figure 3.**
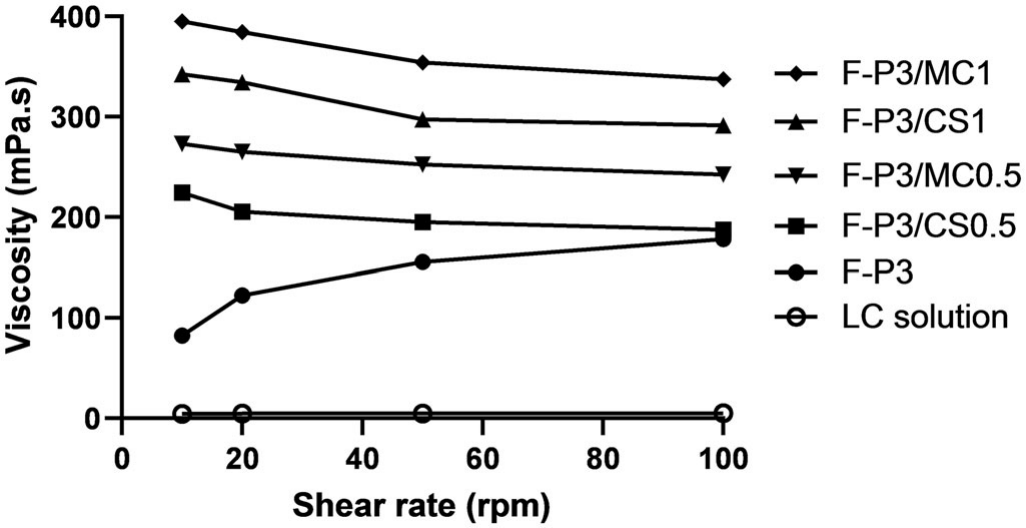
Rheological profiles of different methylcellulose (MC)- and chitosan (CS)-poloxamer (P) hybrid gels compared to P gel and LC solution measured at 4 °C, data represent mean ± *SD*.

**Figure 4.**
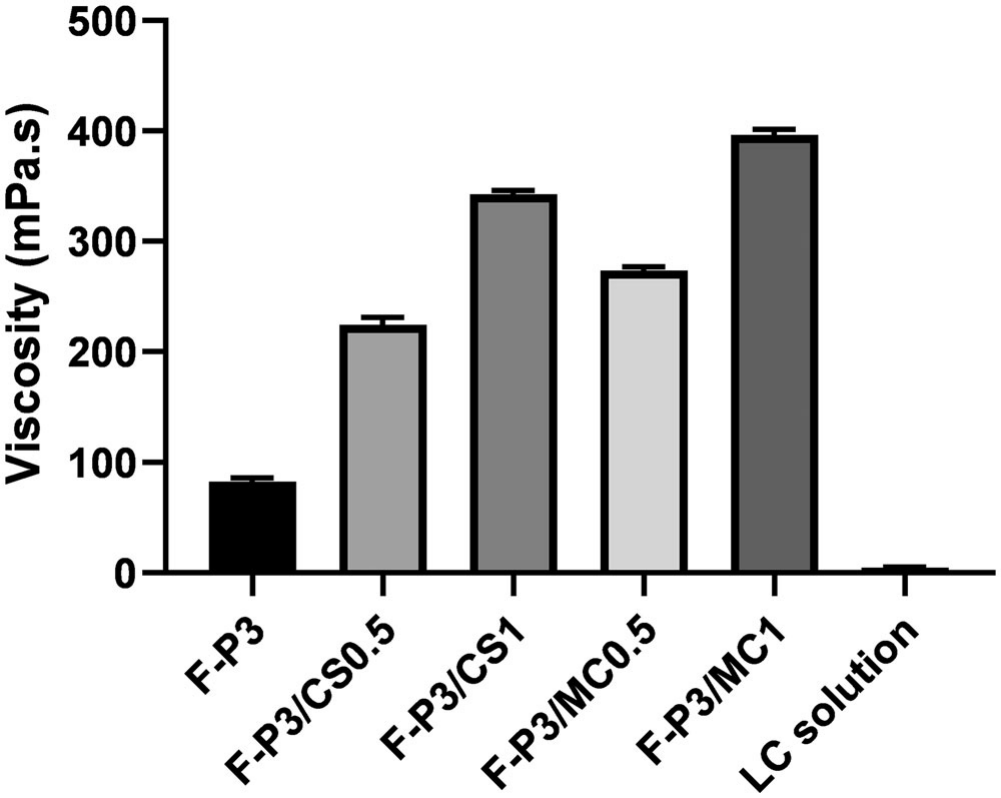
Effect of composition of the gel on viscosity for different methylcellulose (MC)- and chitosan (CS)-poloxamer (P) hybrid gels compared to P gel and LC solution measured at 4 °C, data represent mean ± *SD*.

The rheological characteristics were studied at 4 °C for selected *in situ* gels based on their superior gelation time and temperature as discussed in the previous section. This study was performed to evaluate the viscosity of the formulations while they are in the solution phase before transforming them into a gel. Initial viscosity can give an idea of how the prepared *in situ* gelling formulations could resist initial rapid dilution by resident tears and subsequently promote the prolongation of precorneal residence time (Figures 3 and 4). The more viscous the formulation, the less likely it will undergo dilution by resident tears and more likely it will resist nasolacrimal drainage. Figure 3 and Table 2 show the viscosity values of LC solution (1%) and the selected formulations at different shearing rates (corresponding to different rotational speeds expressed in rpm) measured at 4 °C. Figure 3 shows a typical Newtonian flow behavior for LC solution; the viscosity seems to be constant with the increasing shear rates. On contrary, non-Newtonian flow behaviors were recorded for F-P3 and the other hybrid gels.

**Table 2.** Rheological characteristics, surface tension, contact angle and spreading coefficient measurments for drug solution and L-carnosine *in situ* gels.

*In situ* gels	Viscosity mPaS				Surface tension (γ) (mN/mm)	Contact angle (*θ*°)	Spreading coefficient (S) (mN/mm)
	10 rpm	20 rpm	50 rpm	100 rpm			
F-P3	85.1 ± 8.07	125.5 ± 2.44	160.6 ± 7.00	181.0 ± 3.01	61.52 ± 1.30	45 ± 2.43	−15.08 ± 1.1
F-P3/CS0.5	229.4 ± 11.10	201.4 ± 13.03	191.8 ± 2.76	190.1 ± 11.05	65.59 ± 1.43	41.11 ± 0.88	−16.09 ± 1.43
F-P3/CS1	345.3 ± 8.82	339.3 ± 17.22	301.4 ± 9.22	293.2 ± 7.66	63.79 ± 0.89	46.1 ± 1.33	−19.54 ± 0.91
F-P3/MC0.5	271.4 ± 3.11	267.0 ± 4.33	261.1 ± 3.09	250.2 ± 5.32	58.73 ± 0.88	36.2 ± 0.92	−11.27 ± 1.08
F-P3/MC1	400.3 ± 12.03	389.1 ± 8.12	358.2 ± 17.0	341.4 ± 13.11	57.33 ± 0.92	40.03 ± 0.77	−13.34 ± 1.02
LC solution (1%)	4.8 ± 0.09	4.7 ± 0.08	4.9 ± 0.12	4.5 ± 0.2	70.5 ± 1.63	64.2 ± 2.83	−39.59 ± 1.50

Data are expressed as mean values ± *SD* (*n* = 3).

Figure 4 shows the effect of the composition of the gel formulations on the viscosity.

The viscosity of hybrid gels F-P3/CS0.5 and F-P3/MC0.5 significantly (*p* < .001) increased, compared to poloxamer gels alone. The two-hybrid gel formulations exhibited viscosity values of 2.7 and 3.2 times greater than that for F-P3, respectively. Moreover, these increases were dependent on MC and CS concentrations. For example, both F-P3/CS0.5 and F-P3/MC0.5 exhibited 2.7- and 3.2-times increases of the viscosity, respectively, compared to F-P3. Further, the viscosity enhancement of F-P3/MC/CS systems was 1.5-fold when MC and CS concentrations increased from 0.5 to 1% (Table 2). More interestingly, the non-Newtonian rheological characteristic of F-P3 reversed from shear-thickening to shear-thinning upon the addition of MC and CS. The former behavior could be attributed to micellar entanglements and packing upon increasing the shearing rates. The latter (shear-thinning) behavior of the tested formulations can be considered a desirable rheological characteristic in ocular drug delivery settings. This is because such features could offer less interference with blinking and more comfort to the eye compared with formulations having a shear-thickening behavior (Greaves et al., 1992; Cao et al., 2010).

### Texture analysis of in situ gel formulations

This experiment is performed to understand the mechanical properties of the investigated systems. More specifically, hardness, cohesiveness, and adhesiveness were recorded for the prepared *in situ* forming gels. These properties could simulate certain sensory parameters *in vivo*, and hence help develop an ocular dosage form that offers better patient compliance (Gratieri et al., 2011).

The hardness is a measure of the force required to produce gel deformation. Significantly lower hardness values were recorded for the selected *in situ* gels at 4 °C (which are actually viscous liquids at this temperature) compared to those measured after gelation at 35 °C (Table 3). Instead, the addition of additives like CS, MC did not produce noticeable changes in hardness at 4 °C. However, the hardness reduced to almost half upon addition of CS at 0.5% in the gel state at 35 °C. Raising CS concentration to 1% led to a substantial recovery of the overall hardness of the mixed system when compared to the F-P3 gel. On the contrary, the addition of MC showed concentration-dependent increases in hardness (Table 3). A previous study reported relatively very low hardness for chitosan gel (44.6 g), compared to poloxamer 407 gel (753 g) measured at room temperature (Hurler et al., 2012). Therefore, hybrid gels of CS and P can understandably result in low hardness for the formed hybrid gels, compared to poloxamer 407 alone.

**Table 3.** Mechanical properties (hardness, cohesiveness, adhesiveness), mucoadhesion force, and work of adhesion of some selected L-carnosine *in situ* gels.

*In situ* gels	Temperature (4 °C)			Temperature (35 °C)			Mucoadhesion	
	Hardness (mN)	Cohesiveness (mN·mm)	Adhesiveness (mN·mm)	Hardness (mN)	Cohesiveness (mN·mm)	Adhesiveness (mN·mm)	Adhesion force (mN)	Work of adhesion (mN·mm)
F-P3	17.3 ± 0.2	152.5 ± 3.2	86.1 ± 2.0	190.3 ± 10.1	776 ± 2.1	911 ± 2.9	110 ± 5.5	210 ± 15.5
F-P3/CS0.5	22.6 ± 1.6	115.4 ± 3.2	33.1 ± 3.9	101.3 ± 2.7	411 ± 3.5	836.2 ± 5.9	136 ± 8.5	285 ± 7.5
F-P3/CS1	24.1 ± 1.3	270.4 ± 2.9	39.5 ± 1.9	183.4 ± 1.6	654 ± 6.1	1014 ± 8.8	170 ± 12.5	345 ± 4.5
F-P3/MC0.5	17.1 ± 2.1	116.4 ± 0.9	30.1 ± 6.3	280 ± 3.4	1037 ± 7.9	1210 ± 6.0	107 ± 10.5	170 ± 6.5
F-P3/MC1	23.1 ± 1.1	121 ± 6.3	41.1 ± 2.8	326.1 ± 6.4	1193 ± 11.2	1498 ± 10.4	87.5 ± 4.8	115 ± 2.5

Data are expressed as mean values ± *SD* (*n* = 3).

At a temperature of 35 °C, the formulation is expected to be in the gel state; yet it is desirable that the formulation possesses an appreciable hardness (resistance to deform) to withstand tear dilution and nasolacrimal drainage (Ferrari et al., 1994).

The data presented in Table 3 shows that the hardness values of the gel preparations were comparatively high when measured at 35 °C with respect to those at 4 °C. For example, the hardness values at 4 and 35 °C for F-P3/CS0.5 were 22.6 ± 1.6 and 101.3 ± 2.7 mN, respectively. These results correlate well with the viscosity data. The same results apply to those gel formulations containing different concentrations of MC, where the hardness of the formulations (at 35 °C) increased when MC concentration increased (Table 3). The experimental data further supported our initial hypothesis that both CS and MC have promoted further association and entanglement of P chains, thereby dramatically increasing the hardness of the formulations when the polymer is in its ordered state at 35 °C.

Adhesiveness is a measure of the work necessary to detach the probe from the sample (Xu et al., 2014). Table 3, shows the adhesiveness of different *in situ* gel formulation either alone or in the presence of the additives used. The adhesiveness of the formulation dropped significantly when the formulations were in solution states at 4 °C; on the contrary, slight increases in adhesiveness were reported in gel states with the addition of MC and CS.

The cohesiveness of the *in-situ* gels is an indication of the attractive force between molecules of the investigated systems. Table 3 shows the cohesiveness of the tested *in situ* gels. Similar behaviors were recorded as mentioned with adhesiveness measurements.

### Surface tension, contact angle, and spreading ability of LC-loaded in situ gelling formulations

Table 2 shows *γ*, *θ*, and *S* values for a drug solution and selected formulations. Except for drug solutions, all the prepared *in situ* gels had low surface tension that is compatible with that of the precorneal tear film and exhibited significantly low contact angles (*θ*) and low spreading coefficients (*S*). These results indicate a superior spreading ability of the prepared formulations. These recorded characteristics are favorable features for enhanced performance of newly developed ocular dosage forms.

### *In vitro* ocular irritation studies

#### HET-CAM

**Figure 5.**
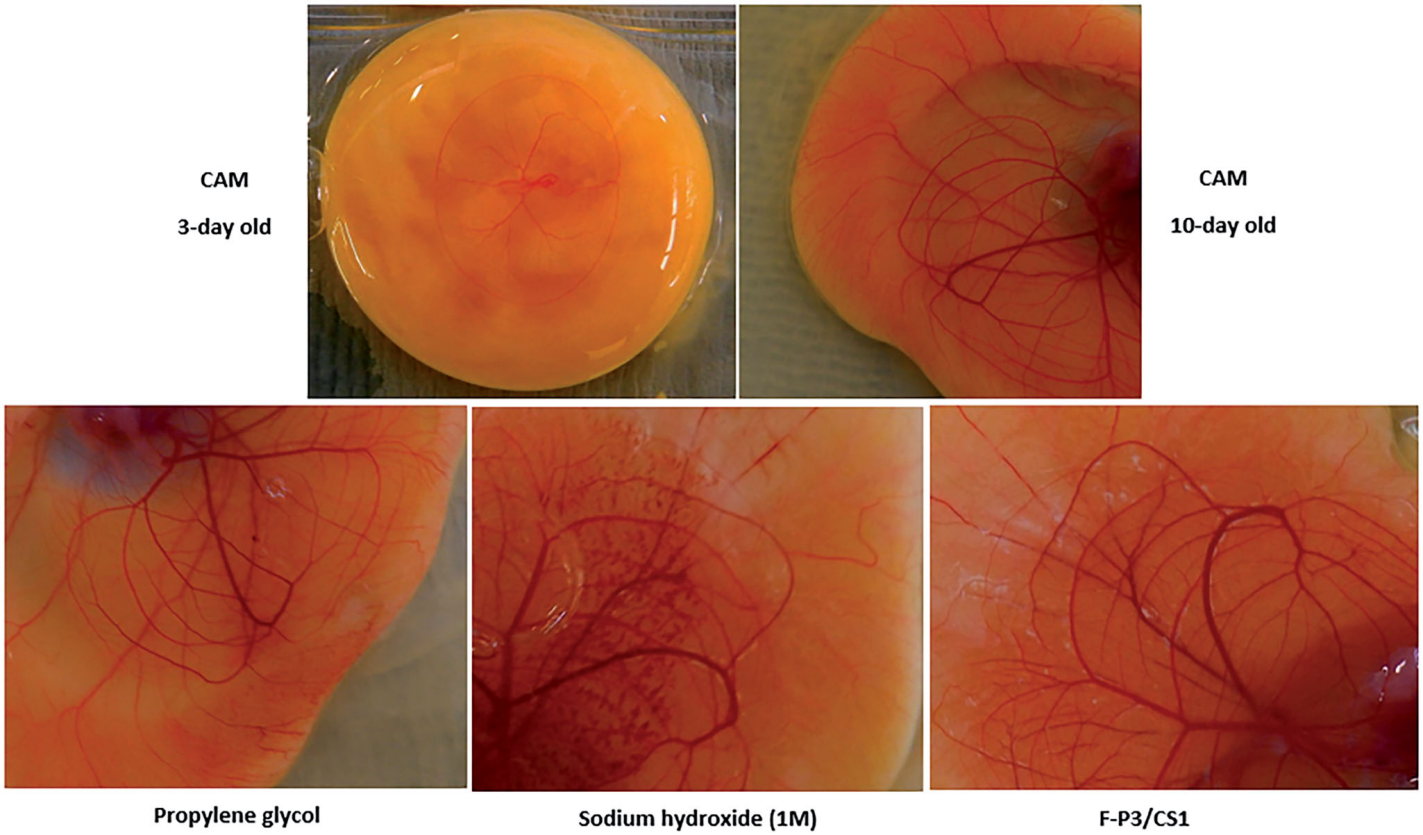
Developmental stages of the CAM and various responses for positive control and F-P3/CS1 *in situ* gel.

**Figure 6.**
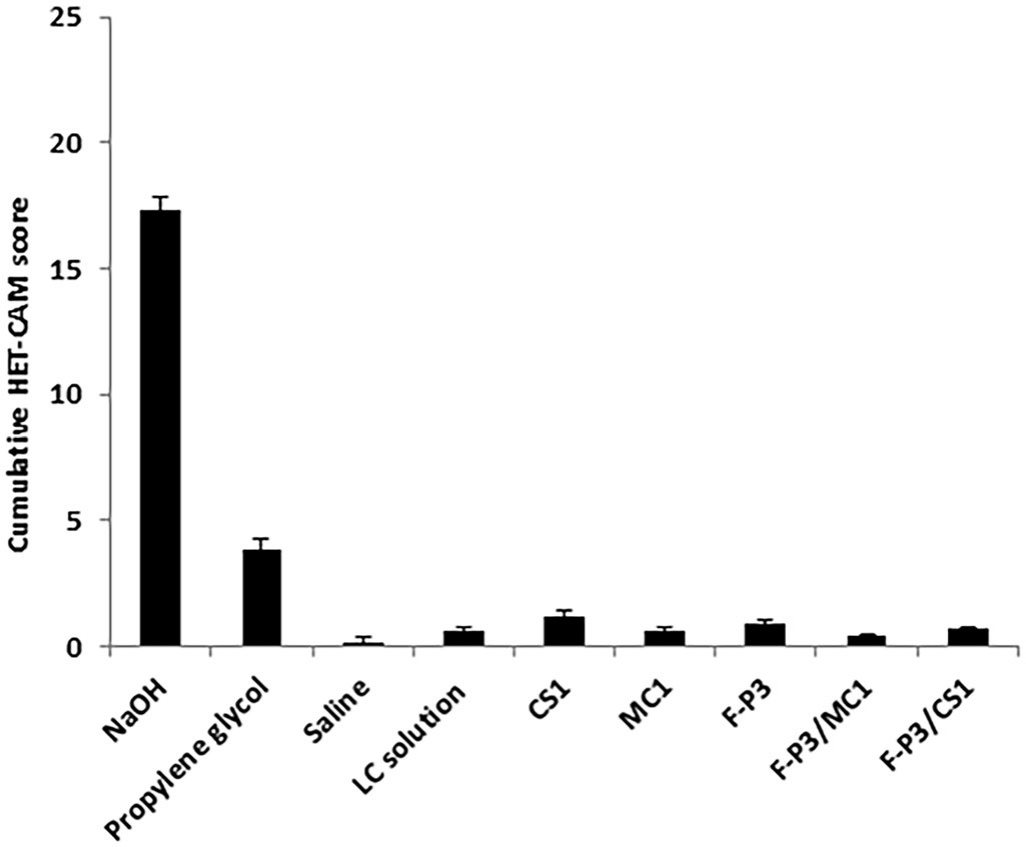
Numerical cumulative irritation scores of HET-CAM after administration of the controls and *in situ* gels.

HET-CAM is a well-accepted *ex vivo* conjunctival irritation model that produces responses (of hyperemia, hemorrhage, and clotting/coagulation) to test substances similar to those involving the conjunctiva of the eye. Figure 5 shows developmental stages of the CAM at 3- and 10-days and different irritant responses to the moderate irritant propylene glycol (PG), a strong irritant (sodium hydroxide 1 M), and F-P3/CS1. Figure 6 shows the cumulative numerical irritation scores of the test substances and controls. Mild-to-moderate hyperemia was observed with administration of PG; intense hyperemia and hemorrhage of the blood vessels and capillaries were observed and recorded with the administration of the corrosive alkali sodium hydroxide. All the tested *in situ* gels displayed slightly to mild hyperemia. That is why they are interpreted as a none-to-mild irritant (Figure 6). There were no statistical significances (*p* > .05) among the prepared *in situ* gels.

### Mucoadhesion studies

The *in vitro* mucoadhesion characteristics of the selected *in situ* gels were assessed by measuring the force of detachment or adhesion (mN) and work of adhesion (mN.mm) of the porcine mucin disk (mimicking mucin layer in the mucus membrane of the conjunctiva) out of the gel surface. Mucoadhesion is an essential property required to extend ocular residence time and improve ocular bioavailability (Lang et al., 2006).

The results are presented in Table 3. The selected *in situ* gels showed well measurable force and work of adhesions ranging from 87.5 to 170 and 115 to 345 mN·mm, respectively. There was a good correlation between the force of adhesion and work of adhesion. The greater the force of adhesion, the higher values were recorded for work of adhesion for the corresponding gel formulations. The weakest mucoadhesion force and the lowest work of mucoadhesion were recorded for F-P3/MC0.5 and F-P3/MC1 whereas the superior mucoadhesion characteristics were displayed by F-P3/CS0.5 and F-P3/CS1.

F-P3 (poloxamer 407 alone) came in the middle. The addition of the cationic polymer chitosan enhanced the mucoadhesion properties probably due to the electrostatic interactions between the negatively charged mucin and the cationic-CS-based *in situ* gels (Lehr et al., 1992, 1994). These effects were dependent on chitosan concentrations. The higher the chitosan concentration, the stronger the electrostatic interaction and hence the greater mucoadhesion force and work of adhesion. On the contrary, the addition of methyl cellulose offered no observable improvement in mucoadhesion properties of the *in-situ* gels. This is could be ascribed to the non-ionic nature of methylcellulose as well as the weak propensity of MC to form hydrogen bonding with mucin due to the relatively high degree (>30%) of hydroxyl-group methylation. Accordingly, P-CS *in situ* gelling formulations (F-P3/CS0.5 and F-P3/CS1) were selected for further studies.

### SEM

**Figure 7.**
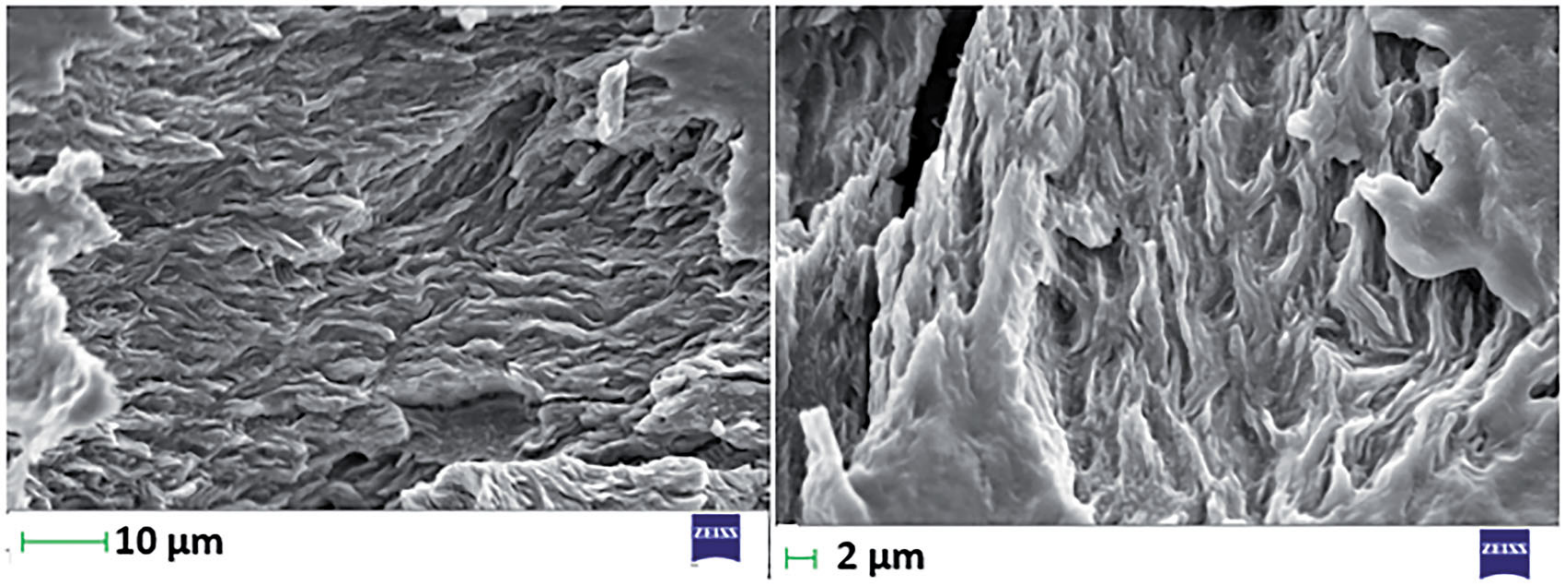
Scanning electron micrographs of F-P3/CS1.

The microstructure of the surface characteristic of F-P3/CS1 was visualized using SEM. Figure 7 shows SE micrographs of F-P3/CS1 at two different magnifications to study the microstructure and surface morphology of the *in-situ* gel. There were no signs of phase/polymer separation. The surface of the gel matrix appeared a corrugated/rough surface.

### *In vitro* L-carnosine release and corneal penetration of the selected in situ gel formulations

**Figure 8.**
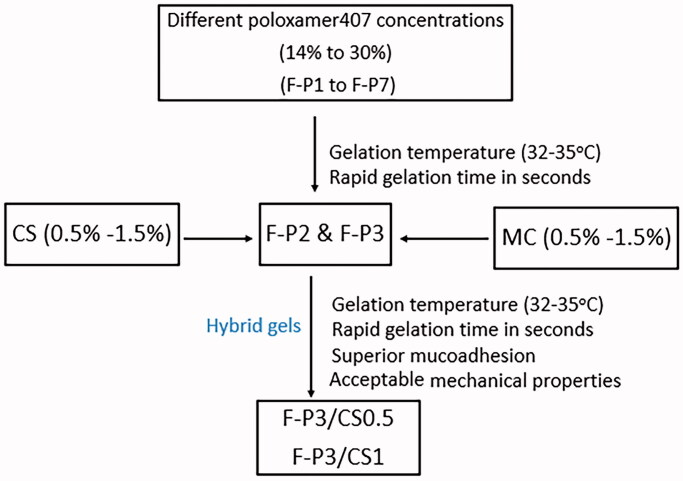
Summary of selection criteria of the optimized formulation.

The selected formulations were chosen for the *in vitro* release study depending on the selection criteria outlined in the flow chart (Figure 8).

**Figure 9.**
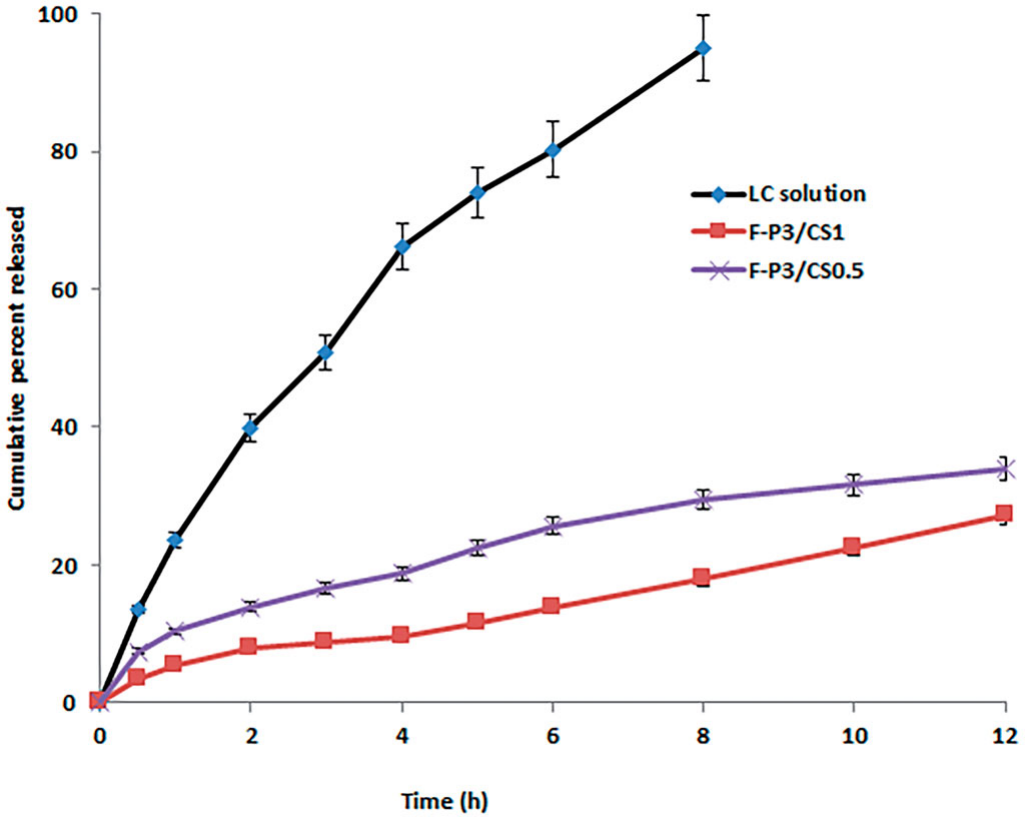
*In vitro* release profiles of L-carnosine from solution and selected *in situ* gelling formulations. Data are expressed as mean values ± *SD* (*n* = 3).

Figure 9 shows *in vitro* release profiles of LC from solution, F-P3/CS0.5 and F-P3/CS1. Markedly prolonged LC release was observed from the selected *in situ* gels with slow but steady drug release profiles, compared to that for the drug solution form. For example, almost complete (>95%) drug release from LC solution was recorded over 8 h compared to only 30 and 18% for F-P3/CS0.5 and F-P3/CS1, respectively. This can be explained on the basis that more extra time was required for drug molecules to diffuse out and escape the extensive gel matrix. On contrary, free drug solutions released LC promptly with faster release rates. For example, the time for 20% drug release was <1, 4, and 8 h for LC solution, F-P3/CS0.5 and F-P3/CS1, respectively. These results also indicated that the release behavior was a CS concentration-sensitive process. The best-fitting release kinetics model was the Higuchi diffusion model with regression coefficient (*R*^2^) >0.99. Similar results were reported with sulforaphane (antiarthritic and immunoregulator drug) loaded into poloxamer-hyaluronic acid hybrid hydrogels. Linear and rapid drug release (complete release in 8 h) from aqueous solution was recorded compared to more sustained release (up to 24 h) from the hybrid gels with a general mechanism of diffusion and erosion (Nascimento et al., 2021).

**Figure 10.**
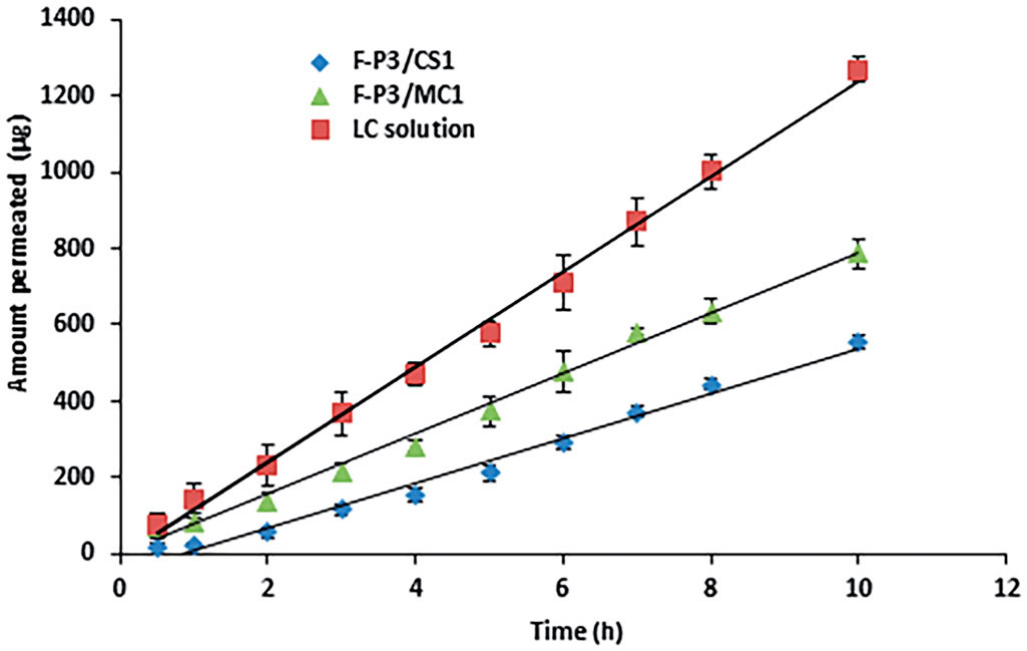
Transcorneal permeation from L-carnosine solution and selected *in situ* gelling formulations. Mean ± *SD*, *n* = 3.

Transcorneal permeation studies for LC solution, F-P3/CS0.5 and F-P3/CS1 were studied using excised bovine corneas. Permeation parameters like the flux and apparent permeability coefficient (*P*_app_) were estimated from the slope of cumulative permeated amounts of L-carnosine *vs.* time (Figure 10) and the results are presented in Table 4. Both flux and *P*_app_ for F-P3/CS0.5 and F-P3/CS1 (*p* < .05) were significantly lower than those for LC solution. This can be ascribed to a high consistency of the two gel forms which markedly slow down drug diffusion through the gel network to eventually give lower transcorneal permeation as expressed by the quantities collected in Table 4.

**Table 4.** Steady-state flux and apparent permeability coefficient (*P*_app_), of LC solution and selected *in situ* gels.

Formulation	Flux (µg/h·cm^2^)	Apparent permeability *P*_app_ × 10^−6^ cm/s
LC solution	171.54 ± 2.03	2.80 ± 1.90
F-P3/CS1	49.96 ± 1.22	0.81 ± 0.57
F-P3/MC1	79.23 ± 1.55	1.29 ± 0.45

Data are expressed as mean values ± *SD*, *n* = 3.

It is worth mentioning that the superior mucoadhesive, spreading ability and viscous gelling features of the prepared *in situ* polymer gels indicate that these adequate formulations hold themselves for a prolonged time on the corneal surface before drainage as it has been previously reported by others (Gratieri et al., 2010, 2011).

### *In vivo* study

Scrapping of the corneal epithelium was induced using ethyl alcohol 70% v/v and utilized as a pharmacodynamic response to compare the corneal wound healing potential of LC when loaded in the optimized *in situ* gelling formulations (F-P3/CS1) in comparison with the control (1% LC solution).

It has been previously shown that the size of the ulcers for eyes exposed to LC solution was markedly less compared to untreated eyes (Babizhayev et al., 2002). Whilst these findings are promising, we hypothesize that incorporating LC in optimized *in situ* gelling formulations would be advantageous.

**Figure 11.**
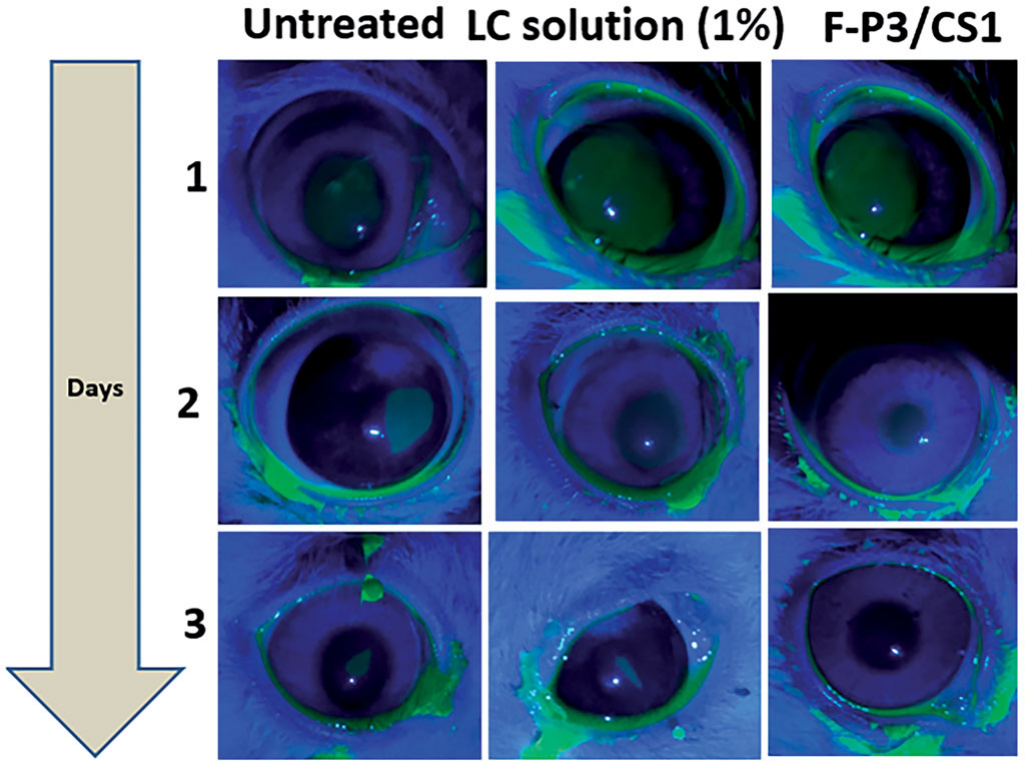
Corneal ulcer visualized under cobalt blue illumination in rabbits’ eyes model.

Figure 11 shows fluorescein-stained rabbit eyes under cobalt blue light to visualize the corneal ulcers. The fastest healing rate was ascribed to the optimized *in situ* gels (F-P3/CS1), whereas a relatively delayed wound repair was experienced by the untreated group. The percentage of changes of ulcer size (%Δ) on day 2 for the untreated, L-carnosine solution and the L-carnosine loaded *in situ* gel formulation was 72% ± 7, 55% ± 5, and 45 ± 3.5, respectively. On day 3, complete healing was recorded for F-P3/CS1 with only 26.5% ± 6 and 16% ± 7 (%Δ) recorded for the untreated, L-carnosine solution groups, respectively. These differences between untreated and treated groups were significant (*p* < .05). This indicates the role of the developed *in situ* gelling formulation (F-P3/CS1) in improving the monitored therapeutics response (corneal wound healing) which is mainly due to a combination of superior mechanical and rheological properties, spreading capacity, mucoadhesive nature as well as its propensity to a prolong LC release.

## Conclusion

Poloxamer-based thermosensitive systems have been investigated for oral and dermal drug delivery systems. However, Ocular application does require optimization with physiology and anatomical barriers of the surface of the eye for optimum drug delivery. Among these properties required to optimize include gelation time and temperature, mechanical, viscosity, mucoadhesive properties, and spreading ability. The idea of combining poloxamer 407 with macromolecular compounds like chitosan and methyl cellulose seems to be promising, as they tend to produce a relatively more viscous gel, superior mucoadhesive, better spreading capacity, rapid gelation, and more importantly slow and steady *in vitro* and *ex vivo* permeation, compared to poloxamer 407 alone and LC solution. Poloxamer 407/chitosan combination has resulted in effective superior mucoadhesive and spreading ability compared to the other poloxamer 407-based formulations. The time required to transform poloxamer-based systems from sol to gel state (gelation time) was significantly reduced by the addition of methylcellulose and chitosan. This is desirable to withstand rapid blinking and nasolacrimal drainage. Gelation temperature was very close to the physiological temperature of the eye surface (32–35 °C). Poloxamer 407 (P) gels with a firm hardness that might interfere with blinking and is likely to produce foreign body sensation. Hybrid gels of CS and P generate gels with significantly less firmness. The optimized F-P3/CS1 formulation showed prolonged trans corneal permeation and enhanced corneal wound healing, hence worthwhile further investigation to develop as an eye drop for topical ocular delivery of LC.

## Data Availability

Data available within the article or its supplementary materials.

## References

[CIT0001] Abdelkader A, Fathalla Z, Moharram H, et al. (2018). 2018 Cyclodextrin enhances corneal tolerability and reduces ocular toxicity caused by diclofenac. Oxid Med Cell Longev 2018:5260976. 29636847 10.1155/2018/5260976 PMC5831967

[CIT0002] Abdelkader H, Wertheim D, Pierscionek B, Alany RG. (2020). Curcumin *in situ* gelling polymeric insert with enhanced ocular performance. Pharmaceutics 12:1158. 10.3390/pharmaceutics12121158 PMC7761359 33260494

[CIT0003] Abdelkader H, Ismail S, Hussein A, et al. (2012). Conjunctival and corneal tolerability assessment of ocular naltrexone niosomes and their ingredients on the hen’s egg chorioallantoic membrane and excised bovine cornea models. Int J Pharm 432:1–10. 22575752 10.1016/j.ijpharm.2012.04.063

[CIT0004] Abdelkader H, Swinden J, Pierscionek BK, Alany RG. (2015). Analytical and physicochemical characterisation of the senile cataract drug dipeptide β-alanyl-L-histidine (carnosine). J Pharm Biomed Anal 114:241–6. 26073114 10.1016/j.jpba.2015.05.025

[CIT0005] Babizhayev MA, Burke L, Micans P, Richer SP. (2009). N-acetylcarnosine sustained drug delivery eye drops to control the signs of ageless vision: glare sensitivity, cataract amelioration and quality of vision currently available treatment for the challenging 50,000-patient population. Clin Interv Aging 4:31–50. 19503764 PMC2685223

[CIT0006] Babizhayev MA, Deyev A, Yermakova VN, et al. (2002). Efficacy of N-acetylcarnosine in the treatment of cataracts. Drugs R D 3:87–103. 12001824 10.2165/00126839-200203020-00004

[CIT0007] Cabana A, Aït-Kadi A, Juhász J. (1997). Study of the gelation process of polyethylene oxidea–polypropylene oxideb–polyethylene oxideacopolymer (poloxamer 407) aqueous solutions. J Colloid Int Sci 190:307–12. 10.1006/jcis.1997.4880 9241171

[CIT0008] Cao F, Zhang X, Ping Q. (2010). New method for ophthalmic delivery of azithromycin by poloxamer/carbopol-based *in situ* gelling system. Drug Deliv 17:500–7. 20500130 10.3109/10717544.2010.483255

[CIT0009] Cao Y, Xu J, Cui D, et al. (2021). Protective effect of carnosine on hydrogen peroxide–induced oxidative stress in human kidney tubular epithelial cells. Biomed Biophys Res Commun 534:576–82. 10.1016/j.bbrc.2020.11.037 33276949

[CIT0010] Carvalho G, Araujo V, Fonseca-Santos B, et al. (2021). Highlights in poloxamer-based drug delivery systems as strategy at local application for vaginal infections. Int J Pharm 602:120635. 33895295 10.1016/j.ijpharm.2021.120635

[CIT0011] Cassano R, Gioia M, Trombino S. (2021). Gel-based materials for ophthalmic drug delivery. Gels 7:130. 34563016 10.3390/gels7030130 PMC8482217

[CIT0012] Collaud S, Peng Q, Gurny R, Lange N. (2008). Thermosetting gel for the delivery of 5-aminolevulinic acid esters to the cervix. J Pharm Sci 97:2680–90. 17828758 10.1002/jps.21181

[CIT0013] Edsman K, Carlfors J, Petersson R. (1998). Rheological evaluation of poloxamer as an *in situ* gel for ophthalmic use. Eur J Pharm Sci 6:105–12. 9795025 10.1016/s0928-0987(97)00075-4

[CIT0014] Fathalla ZMA, Vangala A, Longman M, et al. (2017). Poloxamer-based thermoresponsive ketorolac tromethamine *in situ* gel preparations: design, characterisation, toxicity and transcorneal permeation studies. Eur J Pharm Biopharm 114:119–34. 28126392 10.1016/j.ejpb.2017.01.008

[CIT0015] Ferrari F, Bertoni M, Caramella C, La Manna A. (1994). Description and validation of an apparatus for gel strength measurements. Int J Pharm 109:115–24.

[CIT0016] Florence AT, Attwood D. 1998. Solubility of drugs. In: Physicochemical principles of pharmacy. Bristol: Macmillan Press Ltd.

[CIT0017] Fujimoto K, Minami N, Goto T, et al. (2016). Hardness, cohesiveness, and adhesiveness of oral moisturizers and denture adhesives: selection criteria for denture wearers. Dent J 4:34. 10.3390/dj4040034 PMC5806953 29563476

[CIT0018] Gaafar P, El-Salamouni N, Farid R, et al. (2021). Pegylated liquisomes: a novel combined passive targeting nanoplatform of L-carnosine for breast cancer. Int J Pharm 602:120666. 33933646 10.1016/j.ijpharm.2021.120666

[CIT0019] Gaballa S, El-Garhy O, Moharram H, Abdelkader H. (2020). Preparation and evaluation of cubosomes/cubosomal gels for ocular delivery of beclomethasone dipropionate for management of uveitis. Pharm Res 37:198. 32968868 10.1007/s11095-020-02857-1

[CIT0020] Giuliano E, Paolino D, Fresta M, Cosco D. (2018). Mucosal applications of poloxamer 407-based hydrogels: an overview. Pharmaceutics 10:159. 10.3390/pharmaceutics10030159 PMC6161217 30213143

[CIT0021] Gratieri T, Gelfuso GM, Freitas OD, et al. (2011). Enhancing and sustaining the topical ocular delivery of fluconazole using chitosan solution and poloxamer/chitosan *in situ* forming gel. Eur J Pharm Biopharm 79:320–7. 21641994 10.1016/j.ejpb.2011.05.006

[CIT0022] Gratieri T, Gelfuso GM, Thomazini JA, Lopez RF. (2010). Excised porcine cornea integrity evaluation in an *in vitro* model of iontophoretic ocular research. Ophthalmic Res 43:208–16. 20068374 10.1159/000274494

[CIT0023] Greaves JL, Olejnik O, Wilson CG. (1992). Polymers and the precorneal tear film. STP Pharma Sci 2:13–33.

[CIT0024] Hurler J, Engesland A, Kermany P, Kalko-Basnet N. (2012). Improved texture analysis for hydrogel characterization: gel cohesiveness, adhesiveness, and hardness. J Appl Polym Sci 125:180–8.

[CIT0025] Kurniawansyah I, Rusdiana T, Sopyan I, et al. (2020). *In situ* ophthalmic gel forming systems of poloxamer 407 and hydroxypropylmethyl cellulose mixtures for sustained ocular delivery of chloramphenicole:optimization study by factorial design. Heliyon 6:e05365. 33251348 10.1016/j.heliyon.2020.e05365 PMC7677690

[CIT0026] Lang JC, Roehrs RE, Jani R. 2006. Ophthalmic preparations. In: Beringer P, DerMarderosian A, Felton L, et al., eds. Remington: the science and practice of pharmacy. 21st ed. Philadelphia, PA: Lippincott Williams & Wilkins.

[CIT0027] Lang JC, Roehrs RE, Rodeheaver DP, et al. 2002. Design and evaluation of ophthalmic pharmaceutical products. In: Banker GS, Rhodes CT, eds. Modern pharmaceutics. New York, NY: Marcel Dekker.

[CIT0028] Lehr CM, Bouwstra JA, Schacht EH, Junginger HE. (1992). *In-vitro* evaluation of mucoadhesive properties of chitosan and other natural polymers. Int J Pharm 78:43–8.

[CIT0029] Lehr CM, Lee YH, Lee VH. (1994). Improved ocular penetration of gentamicin by mucoadhesive polymer polycarbophil in the pigmented rabbit. Invest Ophthalmol Vis Sci 35:2809–14. 8188475

[CIT0030] Lim LT, Ah-Kee EY, Collins CE. (2014). Common eye drops and their implications for pH measurements in the management of chemical eye injuries. Int J Ophthalmol 7:1067–8. 25540767 10.3980/j.issn.2222-3959.2014.06.29 PMC4270978

[CIT0031] Litwack G. 2018. Metabolism of amino acids. In: Litwack, G. (ed.) Human Biochemistry. Academic Press, Cambridge, Massachusetts, USA.

[CIT0032] Ljubimov A, Saghizadeh M. (2015). Progress in corneal wound healing. Prog Ret Eye Res 49:17–45. 10.1016/j.preteyeres.2015.07.002 PMC4651844 26197361

[CIT0033] Mendelson SD. 2000. Nutritional supplements and metabolic syndrome. In: Mendelson SD, ed. Metabolic syndrome and psychiatric illness. Academic Press,Cambridge, Massachusetts, USA.

[CIT0034] Nascimento MH, Ambrosio FN, Ferraraz C, et al. (2021). Sulforaphane-loaded hyaluronic acid-poloxamer hybrid hydrogel enhances cartilage protection in osteoarthritis models. Mater Sci Eng C 128:112345. 10.1016/j.msec.2021.112345 34474895

[CIT0035] Quinn PJ, Boldyrev AA, Formazuyk VE. (1992). Carnosine: its properties, functions and potential therapeutic applications. Mol Aspects Med 13:379–444. 9765790 10.1016/0098-2997(92)90006-l

[CIT0036] Sapra P, Patel D, Soniwala M, Chavda J. (2013). Development and optimization of *in situ* periodontal gel containing Levofloxacin for the treatment of periodontal diseases. J Sci Innov Res 2:608–27.

[CIT0037] Turner MD, Sale C, Garner C, Hipkiss AR. (2021). Anti-cancer actions of carnosine and the restoration of normal cellular homeostasis. Biochim Biophys Acta Mol Cell Res 1868:119117. 34384791 10.1016/j.bbamcr.2021.119117

[CIT0038] Ur-Rehman T, Tavelin S, Grobner G. (2011). Chitosan *in situ* gelation for improved drug loading and retention in Poloxamer 407 gels. Int J Pharm 409:19–29. 21335076 10.1016/j.ijpharm.2011.02.017

[CIT0039] Xu X, Shen Y, Wang W, et al. (2014). Preparation and *in vitro* characterization of thermosensitive and mucoadhesive hydrogels for nasal delivery of phenylephrine hydrochloride. Eur J Pharm Biopharm 88:998–1004. 25257714 10.1016/j.ejpb.2014.08.015

[CIT0040] Zarrintaj P, Ramsey J, Samadi A, et al. (2020). Poloxamer: a versatile tri-block copolymer for biomedical applications. Acta Biomater 110:37–67. 32417265 10.1016/j.actbio.2020.04.028

[CIT0041] Zhang K, Shi X, Lin X, et al. (2015). Poloxamer-based *in situ* hydrogels for controlled delivery of hydrophilic macromolecules after intramuscular injection in rats. Drug Deliv 22:375–82. 24601854 10.3109/10717544.2014.891272

